# Piezo1 Channels as Force Sensors in Mechanical Force-Related Chronic Inflammation

**DOI:** 10.3389/fimmu.2022.816149

**Published:** 2022-01-26

**Authors:** Hailin Liu, Jialing Hu, Qingcui Zheng, Xiaojin Feng, Fenfang Zhan, Xifeng Wang, Guohai Xu, Fuzhou Hua

**Affiliations:** ^1^ Department of Anesthesiology, The Second Affiliated Hospital of Nanchang University, Nanchang, China; ^2^ Key Laboratory of Anesthesiology of Jiangxi Province, The Second Affiliated Hospital of Nanchang University, Nanchang, China; ^3^ Jiangxi Province Key Laboratory of Molecular Medicine, The Second Affiliated Hospital of Nanchang University, Nanchang, China; ^4^ Department of Anesthesiology, The First Affiliated Hospital of Nanchang University, Nanchang, China

**Keywords:** Piezo1, chronic inflammation, mechanical force, Ca 2+, pharmacology, MSc

## Abstract

Mechanical damage is one of the predisposing factors of inflammation, and it runs through the entire inflammatory pathological process. Repeated or persistent damaging mechanical irritation leads to chronic inflammatory diseases. The mechanism of how mechanical forces induce inflammation is not fully understood. Piezo1 is a newly discovered mechanically sensitive ion channel. The Piezo1 channel opens in response to mechanical stimuli, transducing mechanical signals into an inflammatory cascade in the cell leading to tissue inflammation. A large amount of evidence shows that Piezo1 plays a vital role in the occurrence and progression of chronic inflammatory diseases. This mini-review briefly presents new evidence that Piezo1 responds to different mechanical stresses to trigger inflammation in various tissues. The discovery of Piezo1 provides new insights for the treatment of chronic inflammatory diseases related to mechanical stress. Inhibiting the transduction of damaging mechanical signals into inflammatory signals can inhibit inflammation and improve the outcome of inflammation at an early stage. The pharmacology of Piezo1 has shown bright prospects. The development of tissue-specific Piezo1 drugs for clinical use may be a new target for treating chronic inflammation.

## Introduction

The human body is permanently exposed to mechanical forces, either passively applied or generated inside cells (1, 2). Cells can sense whether the mechanical stress of the microenvironment changes and can adapt to altered mechanical demands. Most physiological processes are related to mechanical force, which is also one of the initiating factors of tissue damage and inflammation (3). Mechanical force-related inflammation is caused by mechanical force damage to tissues. In general, mechanical forces can be divided based on the tissue bed or the type of force, such as hemodynamic, stretch, and stiffness forces. Inflammation-induced by different mechanical forces has different pathological processes. Generally speaking, when destructive mechanical force is applied to cells, in addition to direct mechanical force damage, mechanical force induces cells to secrete proinflammatory factors and cause indirect damage. When inflammatory factors stimulates factors, local blood vessels experience transient constriction followed by vasodilation through a nerve reflex, increased vascular permeability, and leakage of intravascular fluid through the vessel wall to the outside of the vessel. Inflammatory mediators can induce immune cells to adhere and migrate from the vascular endothelium to the injured site, resulting in inflammatory cell infiltration. Inflammatory cells remove necrotic tissue cells on the one hand and damage normal tissue on the other. Under the stimulation of inflammatory factors and tissue disintegration products, the corresponding growth factors are released, the number of proliferating cells increases locally in inflammation, and the inflammation has turned into a chronic process (4).

Normal inflammation is time-limited as an adaptive defense response of the body, occurring when the injury factor is present and disappearing when the injury is removed. If an acute mechanical injury is encountered, the production of inflammation in the organism is localized, transient, and belongs to acute inflammation (5). Harmful mechanical force signals persist and damage tissues leading to chronic inflammatory diseases. Such as mechanical pressure overload leading to osteoarthritis and lumbar degeneration (6, 7), disturbed haemodynamic shear forces damage vascular endothelial cells and ultimately lead to atherosclerosis (8), fat inflammation and insulin resistance induced by fat cell enlargement eventually progress to obesity and diabetes (9), overload of myocardium induces myocardial inflammation, which leads to long-term myocardial fibrosis and cardiac hypertrophy, etc. (10). The most critical features of chronic tissue inflammation are infiltration of inflammatory cells (such as lymphocytes, plasma cells, and monocytes) within the lesion, tissue destruction, and often more pronounced proliferation of fibrous connective tissue, blood vessels, and epithelial, glandular, and parenchymal cells to replace and repair the damaged tissue. Active inflammation, tissue destruction, and repair responses coincide in chronic inflammation. The ultimate harm of chronic inflammation is mainly the damage to essential organs such as the brain, heart, kidney, etc. It is easy to cause disability, affects labour ability and quality of life, and costly medical expenses, which increases society and families’ economic burden (11).

**Figure 1:**
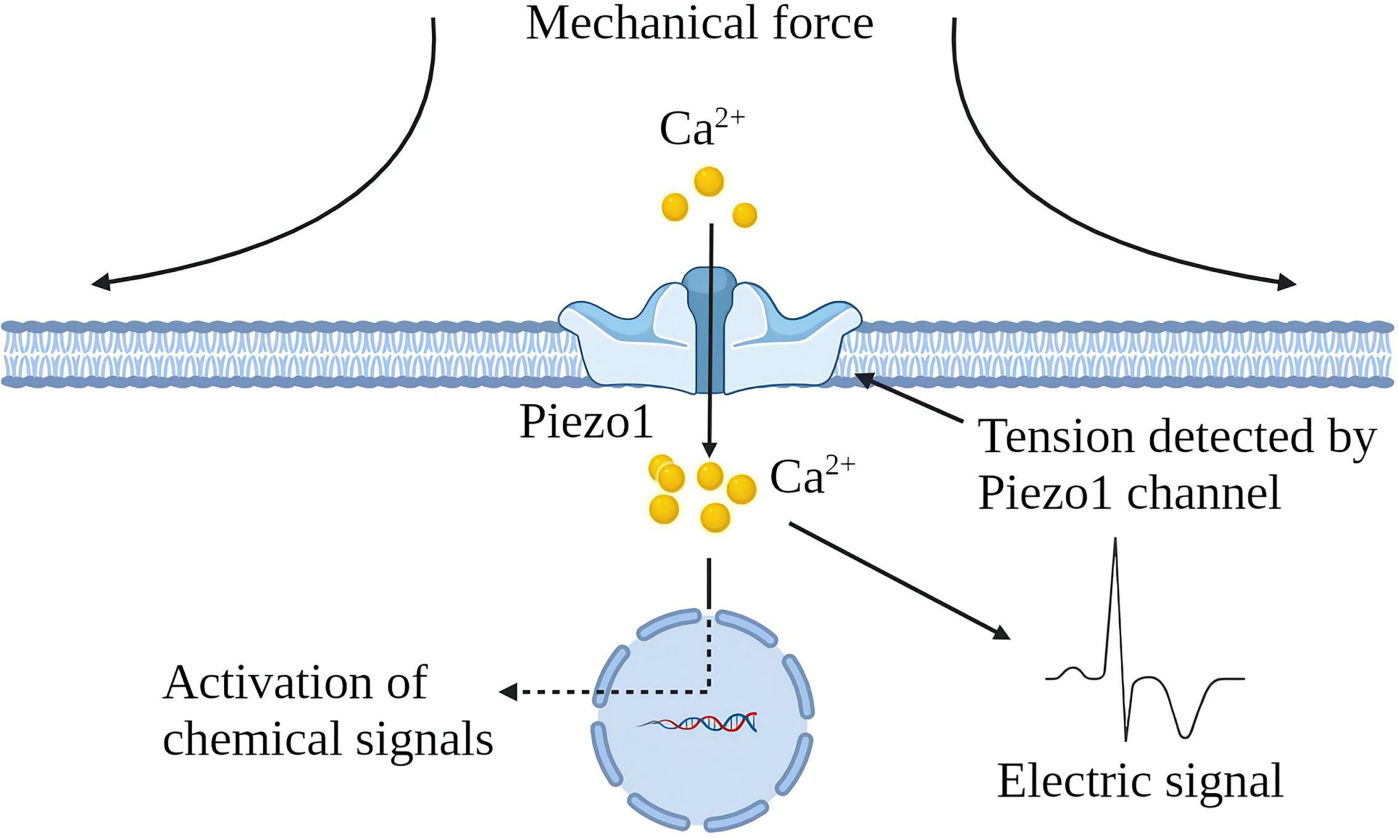
Schematic diagram of Piezo1 channel activation by mechanical force. The Piezo1 channel is a trimeric structure located in the plasma membrane. The mechanical force exerted on the cell membrane causes the opening of the Piezo1 channel, leading to the influx of extracellular Ca^2+^ and transducing mechanical signals into electrical and chemical signals in the cell.

Various mechanical factors can induce chronic inflammation, but cells can perceive noxious stimuli through mechanical transduction (12). All cells exhibit mechanical sensitivity and convert mechanical signals into electrical or chemical signals (13). Piezo1 channel is a newly discovered mechanically sensitive ion channel (MSC), an effective mechanical sensor for cells (14). The latest data shows that the Piezo1 channel is a trimeric structure. In plan view, it looks like a propeller blade or three-pointed tooth, with an ion penetration hole in the middle and a cap on the top [CED (C-terminal extracellular domain)] (15). In a lateral view, the Piezo1 channels distributed on the cell membrane and the lipid membranes surrounding the channels interact to form a dome-like structure and use the trimeric structure described above to mediate mechanical force transmission by a lever principle mechanism. This unique feature is considered essential for force sensing (16). Piezo1 is embedded in the lipid bilayer, which is sensitive to local and global pressure in the bilayer. The Piezo1 channel is mainly located in the plasma membrane. It has been reported to be located in the endoplasmic reticulum and the cytoplasmic compartment, and nuclear envelope near the nucleus (17–21). Piezo1 enables cells to sense various “outside-in” and “inside-out” mechanical forces, including radial pressure, membrane stretching, compression, shear stress, matrix stiffness, ultrasound, matrix nano topology, and osmotic pressure (22). When mechanical stimulation hits the cell membrane, the stress will be distributed to all components, including the double layer, cytoskeleton (CSK) and extracellular matrix (ECM), which converge on the Piezo1 channel and induce the Piezo1 channel from a closed state turn to an open state. The Piezo1 channel allows Ca^2+^, K^+^ and Na^+^ plasma to flow when it is open (**Figure 1**). Piezo1 regulates various functions such as protein synthesis, secretion, migration, proliferation and apoptosis under mechanical pressure (23).

**Figure 2:**
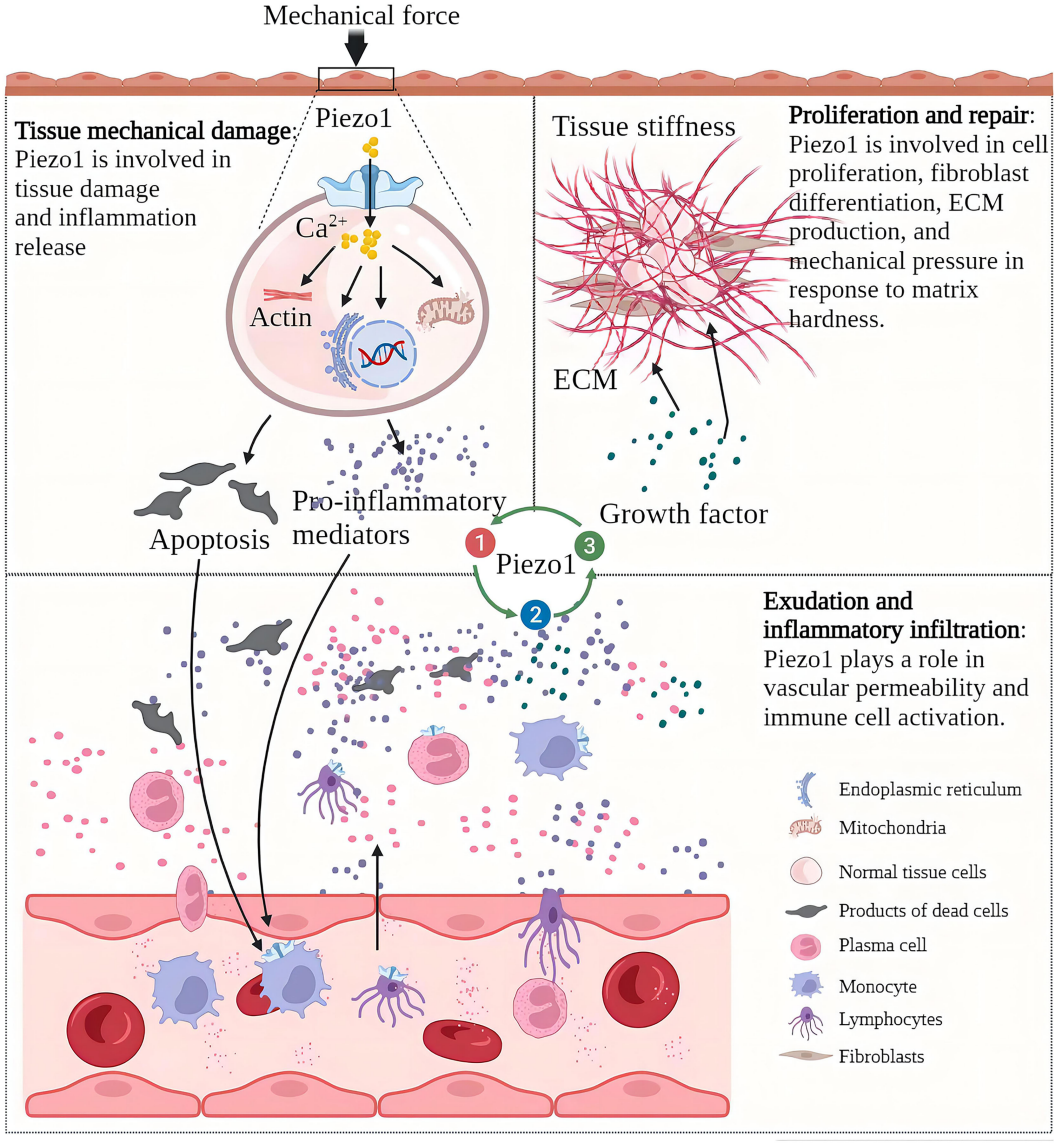
Piezo1 is involved in various pathological chronic inflammation processes and acts as a sensor and effector of mechanical stress within tissues in inflammation. Chronic inflammation is characterized by tissue injury, inflammatory infiltration, and proliferative repair. The Piezo1 channel transduces mechanical forces into intracellular inflammatory signals to induce injury and inflammation, and the development of inflammation alters the mechanical properties of tissues. Piezo1 senses changes in mechanical stress in the local environment and regulates the progression of chronic inflammation.

After mechanical stress damages the tissue and induces tissue inflammation, the progress of inflammation is often accompanied by changes in the mechanical properties of the tissue (24). For example, if edema and necrosis occur after substantial organ injury, the local tissue will relax, and the pressure will decrease (25). If the exudate in the alveoli replaces the air, the density increases, and the pressure increases (26). If the proliferation is based on scar tissue rather than healthy epithelial tissue, there is an increase in local density and increased tissue rigidity (27). Immune cells “squeeze out” from blood vessels and migrate to inflammation sites also show a significant degree of mechanical plasticity (28). As a component of cellular perception of mechanical forces, Piezo1 senses mechanical stress to initiate inflammation and senses changes in local mechanical stress in inflamed tissues and participates in the development of inflammation. As a mechanosensor, Piezo1 senses mechanical forces and initiates inflammation. It also senses changes in local mechanical stress in inflamed tissues and participates in the development of inflammation (29). In acute inflammation, the destructive mechanical forces are released, the inflammatory necrosis clears, and a new balance of Piezo1 function on the cells is reached. The uncontrolled function of Piezo1 in tissues is an essential factor in the development of acute inflammation to chronic. In osteoarthritis, Piezo1 induces cartilage apoptosis and inflammation, inflammatory exudation leads to increased intra-articular interstitial fluid and increased intra-articular pressure, and the increased pressure initiates apoptotic and inflammatory programs (30, 31). In myocardial fibrosis, increasing atrial fibroblast load or heart stretching can activate Piezo1 to increase Ca^2+^ Inflow and promote inflammation and the proliferation and fibrosis of fibroblasts (32). Myocardial fibrosis reduces cardiac compliance and increases the cardiac load, further exacerbating fibrosis. The increase of ECM in the process of lung injury repair will increase the stiffness of the tissue, and the increase of the tissue stiffness will activate the Piezo1 on the fibroblasts, promoting the proliferation of fibroblasts, and the production of ECM components, enhancing and accelerating lung fibrosis (33). In the process of chronic inflammation, Piezo1 perceives changes in tissue “homeostasis” and dysfunction of tissue Piezo1 often accelerates the development of chronic inflammation (**Figure 2**).

**Figure 3:**
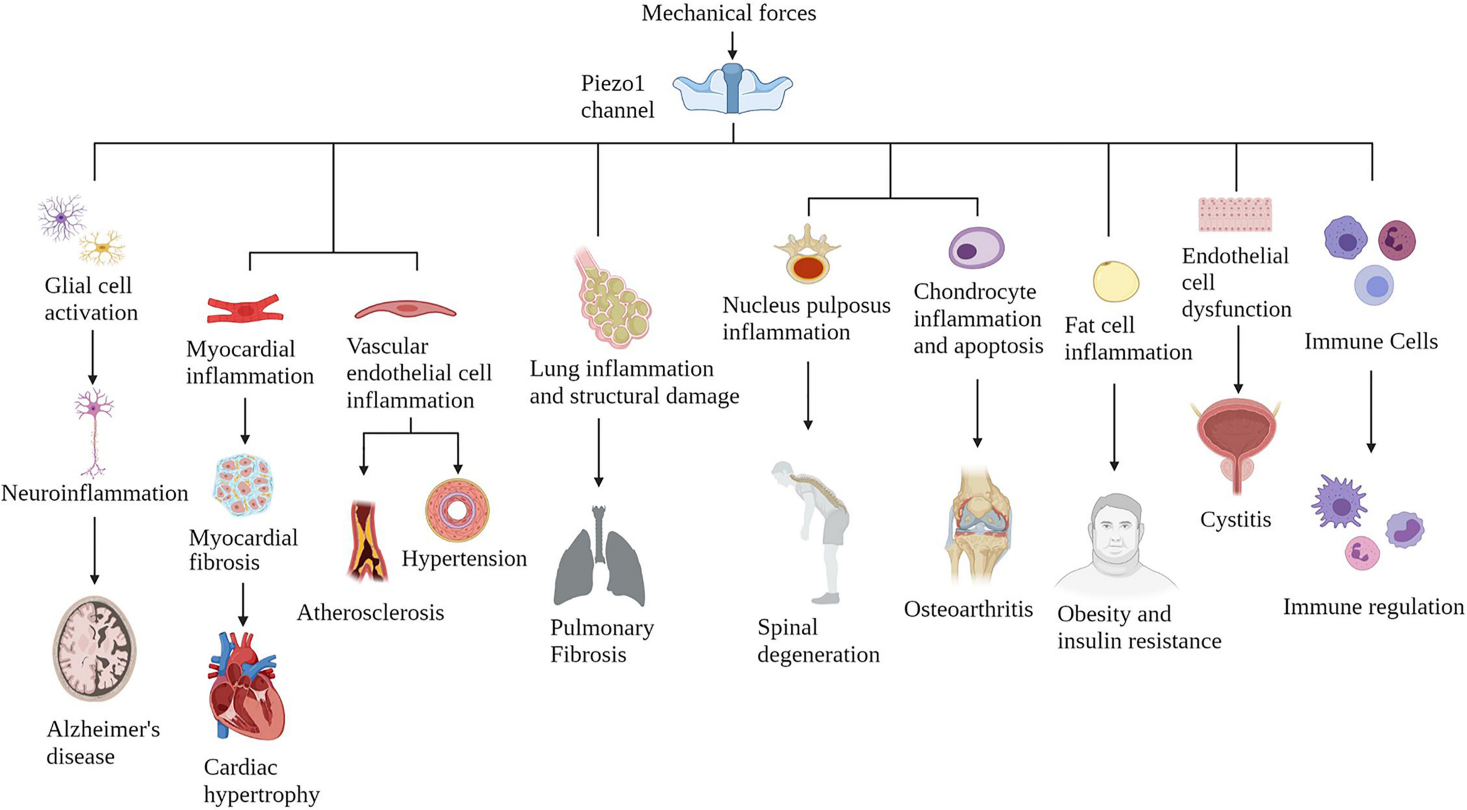
The Piezo1 channel responds to various mechanical force activation and induces chronic inflammatory diseases in multiple systems. The Piezo1 channel transduces mechanical stimulation into an intracellular signal cascade, leading to tissue damage and inflammation.

Piezo1 is widely expressed in various mechanically sensitive cells, and Piezo1 expression has been upregulated in various chronic inflammatory tissues (34) (**Figure 3**). Here, we collate the evidence that Piezo1 is involved in a variety of chronic inflammatory diseases, highlight how Piezo1 initiates inflammatory responses in mechanotransduction. Piezo1 can play a vital role in developing chronic inflammatory diseases by transducing mechanical stimuli into intracellular pro-inflammatory signals in response to various transient or sustained injurious mechanical signals. Based on the role of Piezo1 in inflammatory signal transduction, a large amount of evidence shows that inhibiting Piezo1 can block inflammation in the early onset of chronic inflammatory diseases.

## Piezo1 Channel-Mediated Chronic Inflammation

### Alzheimers (AD)

Both central neurons and glial cells have a high degree of mechanical sensitivity and can respond to changes in the stiffness of the surrounding environment and react quickly (35, 36). Normal brain tissue cells are in a quiet environment rich in polysaccharides, and changes in the stiffness of the brain matrix will affect the development and neurophysiology of brain cells (37). β-amyloid(Aβ) plaque deposits and neuroinflammation are signs of AD, and increased brain stiffness is detected with Aβ plaque deposits (38). β-amyloid plaques are very brittle and rigid in structure (stiffness is about 3×10^9^Pa), and the surface morphology of the brain tissue deposited by the plaques will become rougher. Astrocytes are the most abundant type of glial cells in the brain, and they play an established role in maintaining neuronal function (39). Peripheral infection and ageing can upregulate the expression of Piezo1 in reactive cortical astrocytes (36). Increased expression of Piezo1 in reactive astrocytes exposed to Aβ is not detected in non-AD brains (36). In the brain of AD, astrocytes are more reactive and release pro-inflammatory cytokines more. Piezo1 may potentially regulate the signal transduction of reactive astrocytes, thereby affecting the phenotype of astrocytes. Rough Aβ plaques also induce dynamic changes in astrocyte-neuron interactions, affecting neurotransmission, signal gradients, and the interconnection between synapses (40).

**Figure 4:**
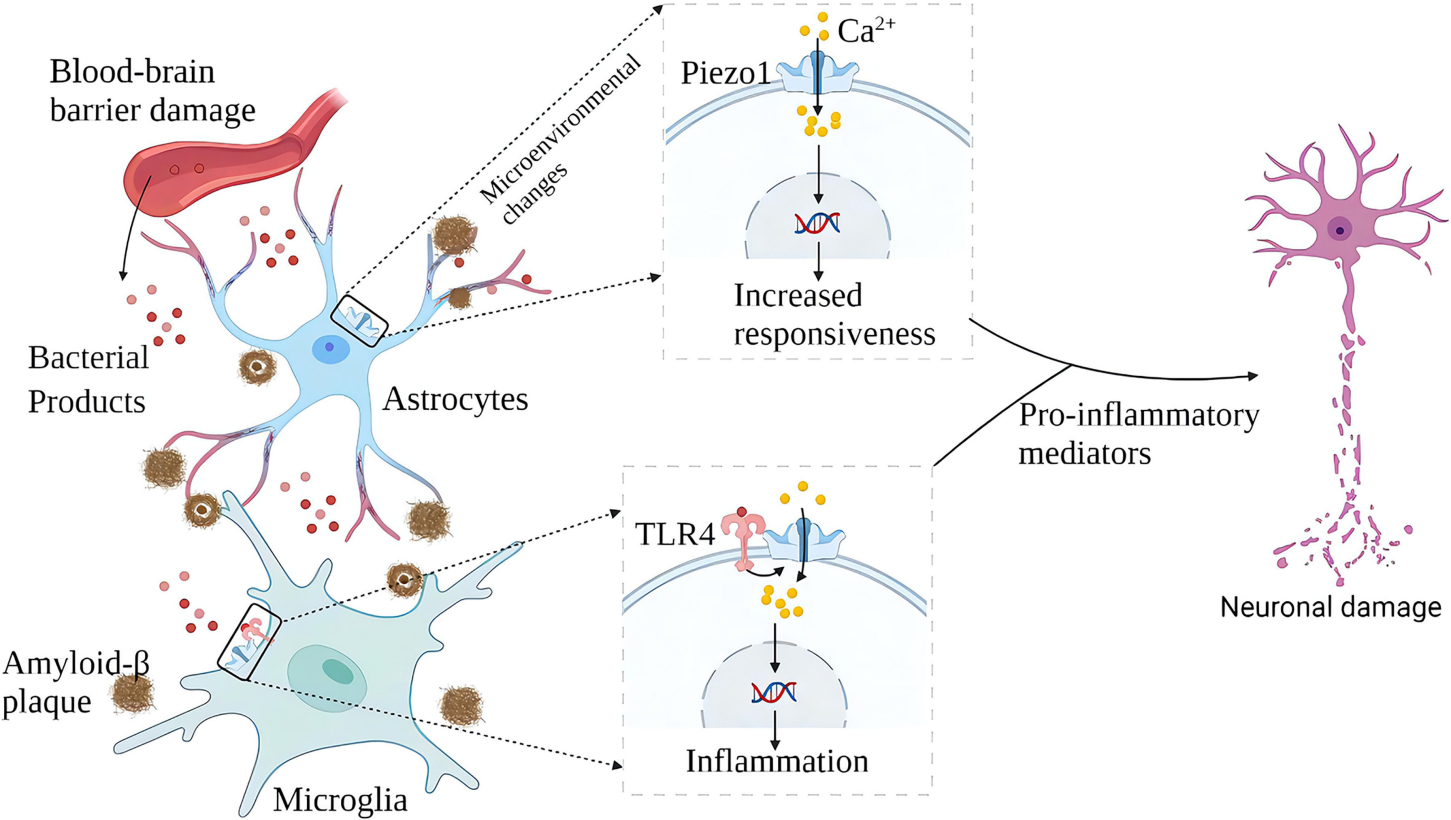
Schematic diagram of the role of Piezo1 in AD. In AD, plaque deposited brain tissue has increased stiffness, increased expression of Piezo1 in astrocytes and microglia exposed to amyloid plaques, and increased inflammatory reactivity. Upregulation of Piezo1 in cerebral vascular endothelial cells induced by various mechanical factors damages the vascular endothelium, leading to increased blood-brain barrier permeability and more bacterial products entering the brain. Bacterial products such as LPS activate TLR4 to initiate microglial innate immune responses, and Piezo1 coordinates TLR4 signaling and induces Ca^2+^ inward flow, regulating cellular immune activity. Chronic inflammation and neuronal damage lead to cognitive impairment.

Microglia are the brain’s resident immune cells, and it is currently believed that they originated from monocytes in the bone marrow. In AD brains, microglia accumulate around Aβ plaques and invade their nuclei for phagocytic clearance of Aβ. Aβ stimulates microglia to trigger the release of various pro-inflammatory cytokines, leading to neuroinflammation, neuronal dysfunction, and death. Piezo1 plays multiple roles in the inflammatory activation of microglia. On the one hand, microglia express TLR4, which is an innate immune receptor (41). Aβ and infection-related bacterial lipopolysaccharide (LPS) activates TLR4 while upregulating Piezo1. Piezo1 may coordinate TLR signals and induce Ca^2+^ influx, and the latter may regulate cellular immune activity through activation of the CaMKII-Mst1/2-Rac axis (42). Therefore, Piezo1 may be involved in the innate immune regulation of glial cells (42). On the other hand, stiff amyloid plaques may upregulate Piezo1 in microglia, thereby affecting the immune activity of microglia. *In vitro*, microglia cultured on stiffness gradient hydrogels migrate to harder areas and are more reactive in harder areas (43). Piezo1 also regulates microglial function in acute hyperglycemic stress, indicating that Piezo1 plays an essential role in hyperglycemic brain injury (44) .In the AD brain, amyloid deposition and neuroinflammation may form a vicious circle. Aβ plaque deposits harden the brain matrix and pro-inflammatory activation of glial cells. A large number of pro-inflammatory factors aggravate neuronal damage, and nerve damage will further release amyloid (**Figure 4**). Although elevated expression of Piezo1 was not detected in AD brain neurons, María Velasco-Estevez et al. (45) showed that neuronal Piezo1 overactivation induces neural demyelination and nerve damage. Piezo1 inhibits axonal regeneration and affects repair after nerve injury (46). This may partly explain the pathogenesis of AD. The accumulation of Aβ is a long-term chronic process. When the balance between the production and elimination of Aβ is broken, the cognitive ability of patients with a large amount of Aβ accumulation will rapidly decline (47, 48).

### Myocardial Fibrosis and Atherosclerosis

**Figure 5:**
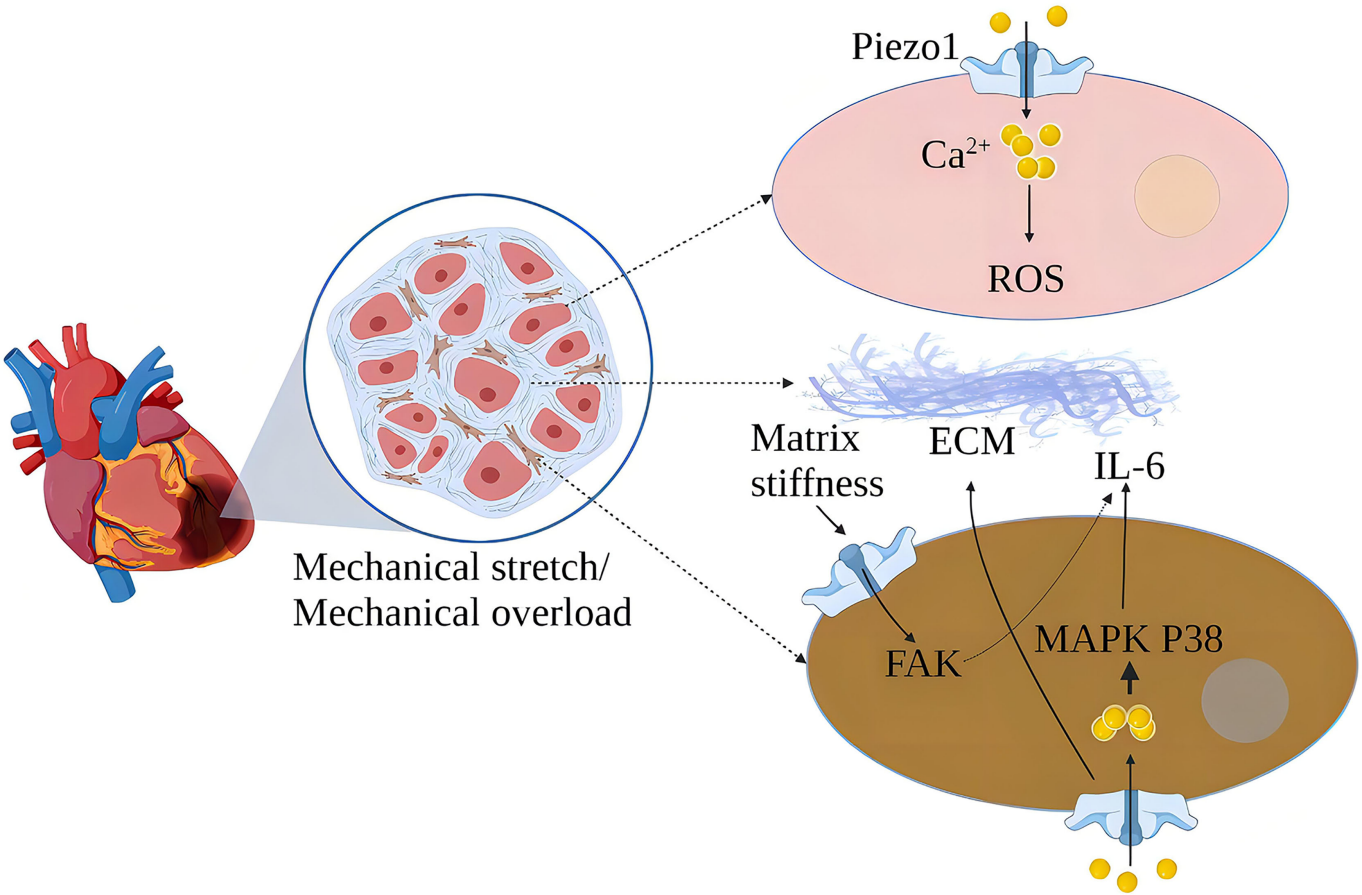
Schematic diagram of the mechanism of action of Piezo1 in myocardial fibrosis. Piezo1 helps cardiomyocytes transduce mechanical stretch into intracellular Ca^2+^ signaling and ROS signaling, and mechanical overload leads to increased Piezo1 expression in cardiomyocytes, ultimately leading to cardiomyopathy. *In vitro*, mechanical stretch-induced fibroblast Piezo1 opening and promoted IL-6 mRNA expression through the MAPKP38 signaling pathway. Mechanical stretch induces fibroblasts to increase ECM secretion, and increased matrix stiffness may further upregulate Piezo1 expression and promote fibrosis progression.

In the cardiovascular system, myocardial contraction pumps blood into the arteries, and blood flow creates friction and pressure on the blood vessels (49). MSCs are widely expressed in the cardiovascular system, sensing changes in mechanical forces and transducing mechanical signals into chemical and electrical signals (50). Piezo1 is one of the cardiovascular sense and plays a crucial role in cardiovascular development, blood pressure regulation, hypertensive vascular remodeling, and other physiology and pathology (51–54). Cardiac fibroblasts play an essential role in the normal physiological function of the heart and its response to damage or stress (55). Piezo1 helps atrial fibroblasts to adapt to changes in the hardness of the surrounding matrix stiffness and modulates fibroblast mechanical properties (56). Mechanical stretching *in vitro* induces activation of atrial fibroblasts, which stimulates the production of ECM and stimulates the production of chemokines, such as monocyte chemoattractant protein (MCP)-1 and MCP-3, and triggers typical inflammatory pathways (57). Increased cardiomyocyte load or cardiac stretch can activate Piezo1 to increase Ca^2+^ influx, leading to increased expression of the inflammatory/mast cell factor IL-6 mRNA. IL-6 is a pleiotropic pro-inflammatory cytokine that promotes the proliferation and fibrosis of cardiac fibroblasts (32). Myocardial fibrosis (MF) reduces cardiac compliance and pumping function (58) (**Figure 5**).

Atherosclerosis (AS) is a multifactorial inflammatory disease, vascular endothelial cells (ECs) injury is the initiating factor of AS. After an endothelial injury, cholesterol and lipids in the blood are deposited under the endothelium and attract monocytes to accumulate, monocytes differentiate into macrophages, phagocytic lipids are transformed into foam cells, and foam cells secrete pro-inflammatory factors (59). Vascular ECs continue to be subjected to the shearing force of the blood flow. In AS, Piezo1 is associated with ECs damage and macrophage activation (60, 61). The damage and anti-damage effects of Piezo1 depend on the type of blood flow signal. Blood flow includes both laminar and turbulent flow. In general, laminar flow leads to NO formation, barrier protection and protect against inflammation, while disturbed blood flow leads to vasoconstriction, permeable barriers, and inflammatory signaling - atherosclerosis develops. ECs in continuous laminar flow is subjected to shear forces only in the direction of the cell axis. In contrast, cells in turbulent blood flow are subjected to forces in a random direction (62). Turbulence activation of Piezo1 channels on vascular endothelial cells induces inflammatory signaling *via* integrin-associated PECAM-1/VE-calmodulin/VEGFR2 and PI-3-kinase-dependent activation, leading to FAK-dependent NF-κB activation, as well as reduced eNOS activation by promoting cAMP degradation *via* activation of phosphodiesterase 4D. NF-κB activation leads to the expression of leukocyte adhesion molecules including VCAM-1 and ICAM-1 and chemokines including CCL2, which promotes the progression of AS (63–65). NF-κB also promotes NLRP3 assembly, caspase-1 triggering, and IL-1β production (66).

**Figure 6:**
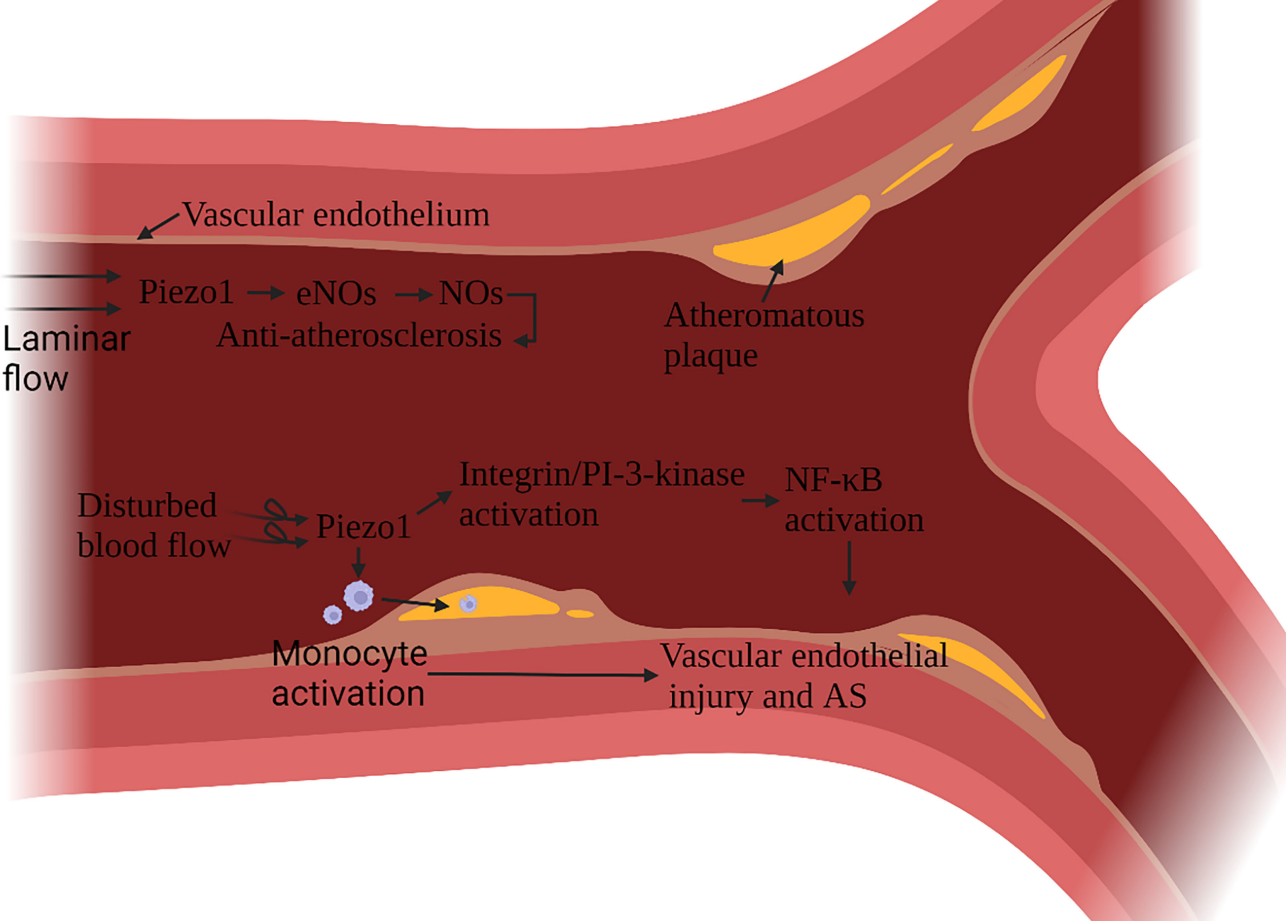
Schematic diagram of the role of Piezo1 in atherosclerosis. Piezo1 on vascular endothelial cells senses the shearing force of blood flow, and laminar flow produces NO through Piezo1, which has the effect of protecting vascular endothelium. Disturbed blood flow shear force activates Piezo1, activates inflammatory pathways through integrin activation and PI3K-related signaling pathways to cause vascular endothelial inflammation, reduces eNOS activation, and induces monocytes to accumulate to the injured site, accelerating the formation of atherosclerotic plaques.

Atherosclerotic plaque leads to arterial stenosis and high blood flow shear stress, which can activate a variety of monocyte activation through Piezo1, promote the adhesion of muscle cells to stimulated endothelial cells, phagocytic activity and low-oxidized density lipoprotein uptake and cytokine expression (67) (**Figure 6**). Stenting to correct disturbed blood flow to laminar flow effectively protects ECs (68). It has been confirmed that laminar flow induces the release of ATP through the Piezo1 channel on the ECs. It then activates the downstream signal mediated by P2Y2/Gq/G11, which further leads to the phosphorylation of AKT and the release of eNOS to participate in blood pressure regulation and anti-atherosclerosis (69). Expanding blood flow channels to reduce high shear stress can also reduce the pro-inflammatory effect of monocytes (67).

Piezo1 is necessary to regulate NO formation, vascular tone, and blood pressure (69). Damage to vascular ECs resulted in vascular sclerosis and increased peripheral resistance, which is one of the essential mechanisms for the occurrence and development of hypertension. IL-6 produced by endothelial cell injury stimulates the synthesis of several acute-phase reactive proteins, including CRP, serum amyloid A, fibrinogen, TNFα and IL-1β. IL-6 can cause ECs dysfunction, increases peripheral vascular resistance and raising blood pressure (70). IL-6 also stimulates the adhesion and aggregation of neutrophils in capillaries, aggravating inflammation and endothelial damage (71). Piezo1 plays an essential role in the remodeling of congenital arteries (72, 73). In addition, Piezo1 is up-regulated in rats with Ang II-induced heart failure, indicating that Piezo1 is also involved in arterial remodeling after injury (74). Excessive activation of Piezo1 in cardiomyocytes leads to excessive ROS and causes cytotoxicity (55). In addition to oxidative stress, ROS can also inactivate the vasodilation function of NO, reducing the amount of NO that can play a role, leading to the consequences of hypertension (75). Piezo1 may also regulate blood pressure by participating in the rat’s motor booster reflex (76).

### Pulmonary Fibrosis

Pulmonary fibrosis is the end-stage changes of a large group of lung diseases characterized by the proliferation of fibroblasts and the accumulation of a large amount of extracellular matrix accompanied by inflammatory damage and destruction of tissue structure. Normal alveolar tissue is damaged and undergoes abnormal repair, leading to abnormal structure (scar formation) (77). Lung tissue is a highly mechanized organ. Piezo1 responds to lung mechanical stress and is involved in the development of pulmonary fibrosis through multiple mechanisms.

Alveolar surfactants play a role in reducing alveolar surface tension and maintaining lung compliance (78). The membrane tension generated by alveolar swelling activates Piezo1 in alveolar type I epithelial cells (AT I), and the Piezo1-mediated rise in Ca^2+^ activates pannexin hemichannels and leads to ATP release. The released ATP stimulates the nearby AT II to release alveolar surfactants (79). High alveolar surface tension leads to alveolar surfactant dysfunction. Alveolar surfactant dysfunction is associated with repeated or continuous damage to the alveolar epithelium and pulmonary fibrosis (80). Animal models of alveolar hypertonicity show different signaling pathways that induce pulmonary fibrosis through a mechanical transduction. These include the Rho/rho-related protein kinase (ROCK), corticotrophin-related transcription factor-A (MRTF-A) and yes-associated protein 1 (YAP)/(transcriptional co-activator with PDZ binding motif) TAZ signaling pathways (81). YAP and TAZ accumulate in cell nuclei exposed to high mechanical stress or undergoing deformation and cytoskeletal tension. YAP/TAZ exerts its pro-fibrosis effect through interaction with nuclear transcription factors and activation of different ECM genes (82). Mechanical ventilation is the primary method for treating acute respiratory distress syndrome (ARDS). Relative to the tiny tidal volume, the excessive expansion of lung volume in mechanical ventilation will cause ventilator-related lung injury (VILI). Piezo1 played a crucial role in VILI after ARDS. Mechanical stretching activates Piezo1 and increases the activity of calcium-dependent calpain to destroy the lung endothelial barrier. Mechanical stretching also induces the activation of Piezo1 channels in AT II. The increase of intracellular Ca^2+^ induces cell apoptosis and abnormal secretion of alveolar surfactant (83), thereby aggravating lung damage and inflammation in patients with ARDS (84). Cyclic stretching activates Piezo1 during mechanical ventilation and affects VILI development by promoting RhoA/ROCK1 signaling. Ionizing radiation upregulates the Piezo1 channel in ATII ​​and regulates the radiation-induced epithelial-mesenchymal transition by forming positive feedback with TGF-β1. The repair of lung tissue after injury increased the stiffness of the extracellular matrix (ECM), leading to the nuclear localization of YAP in fibroblasts, which subsequently showed increased production of ECM components. This may increase the stiffness of the ECM again and start a vicious circle, which may eventually lead to fibrosis.

**Figure 7:**
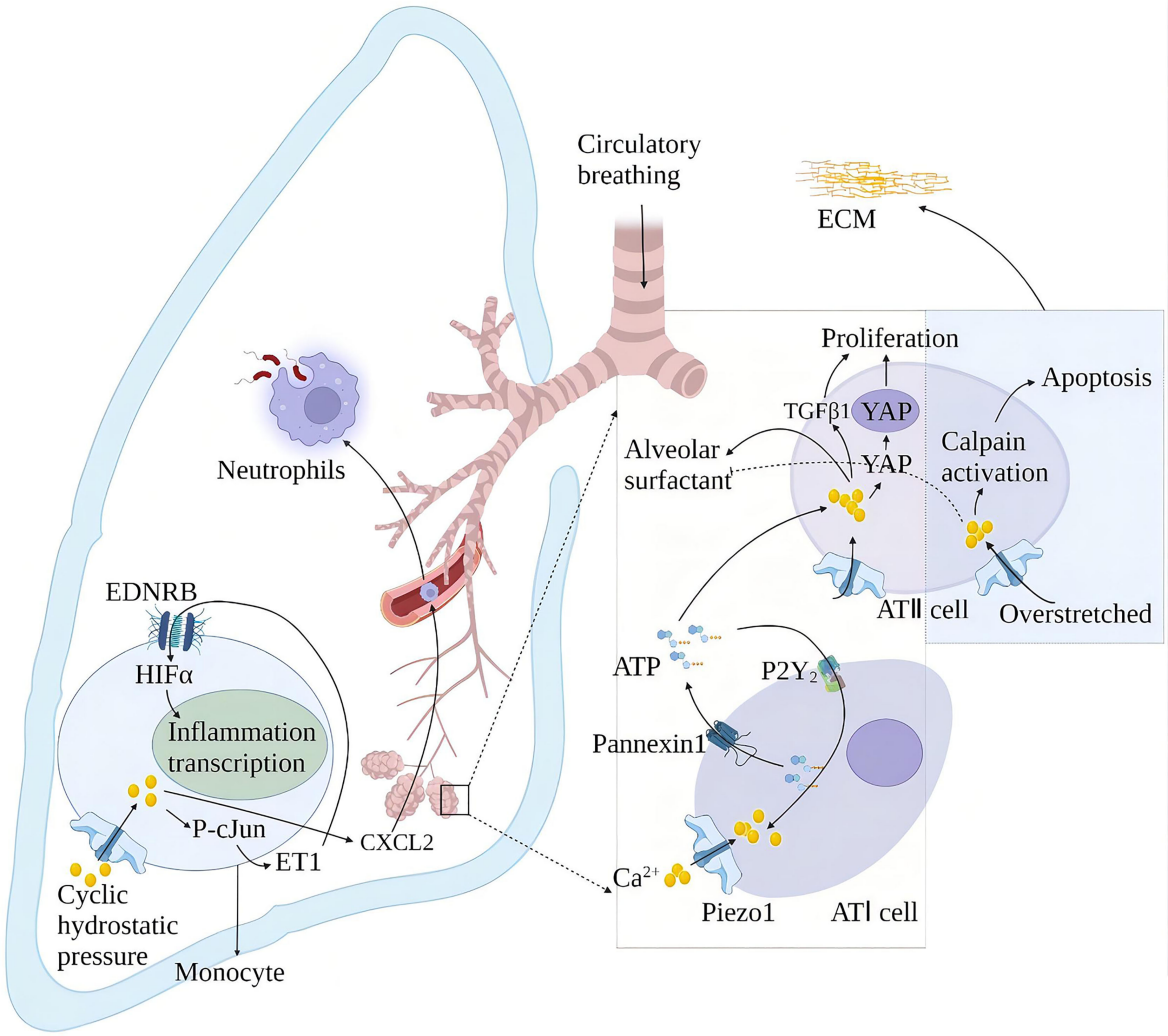
Schematic diagram of the role of Piezo1 in innate lung immunity and pulmonary fibrosis. Periodic hydrostatic pressure in the lungs can activate monocyte Piezo1 to cause Ca^2+^ influx, phosphorylation of transcription factor cJun, and expression of endothelin 1 (ET1), which in turn activates HIF1α *via* the endothelin receptor type B (EDNRB), Thereby promoting the transcription of pro-inflammatory mediator programs. Piezo1 also increased the secretion of the chemokine CXCL2 in monocytes in the lung, inducing neutrophils to migrate from the blood vessels to the lungs to clear bacteria. Piezo1 senses alveolar tone and is involved in the secretion of alveolar surfactant and proliferation of ATII. Alveolar hyperextension leads to reduced secretion of alveolar surfactant and apoptosis of ATII, exacerbating lung injury and inflammation in ARDS patients. Piezo1 upregulation in alveoli induces an epithelial-mesenchymal transition with increased extracellular matrix (ECM) stiffness and promotes pulmonary fibrosis.

Periodic hydrostatic pressure in the lungs can activate monocyte Piezo1 and cause Ca^2+^ influx, phosphorylation of transcription factor cJun, and expression of endothelin 1 (ET1), which in turn activates hypoxia-inducible factor 1α (HIF1α) *via* the endothelin receptor type B (EDNRB), thus facilitating transcription of an effective, prolonged pro-inflammatory mediator program (85). This effect of Piezo1 also increases the secretion of chemokine CXCL2 in monocytes, allowing neutrophils to migrate from the blood to the lungs along the CXCL2 gradient and inducing neutrophils to clear bacteria (86). Knockout of the Piezo1 gene in mice has a protective effect on pulmonary fibrosis induced by bleomycin (85) (**Figure 7**).

### Osteoarthritis and Lumbar Degeneration

**Figure 8:**
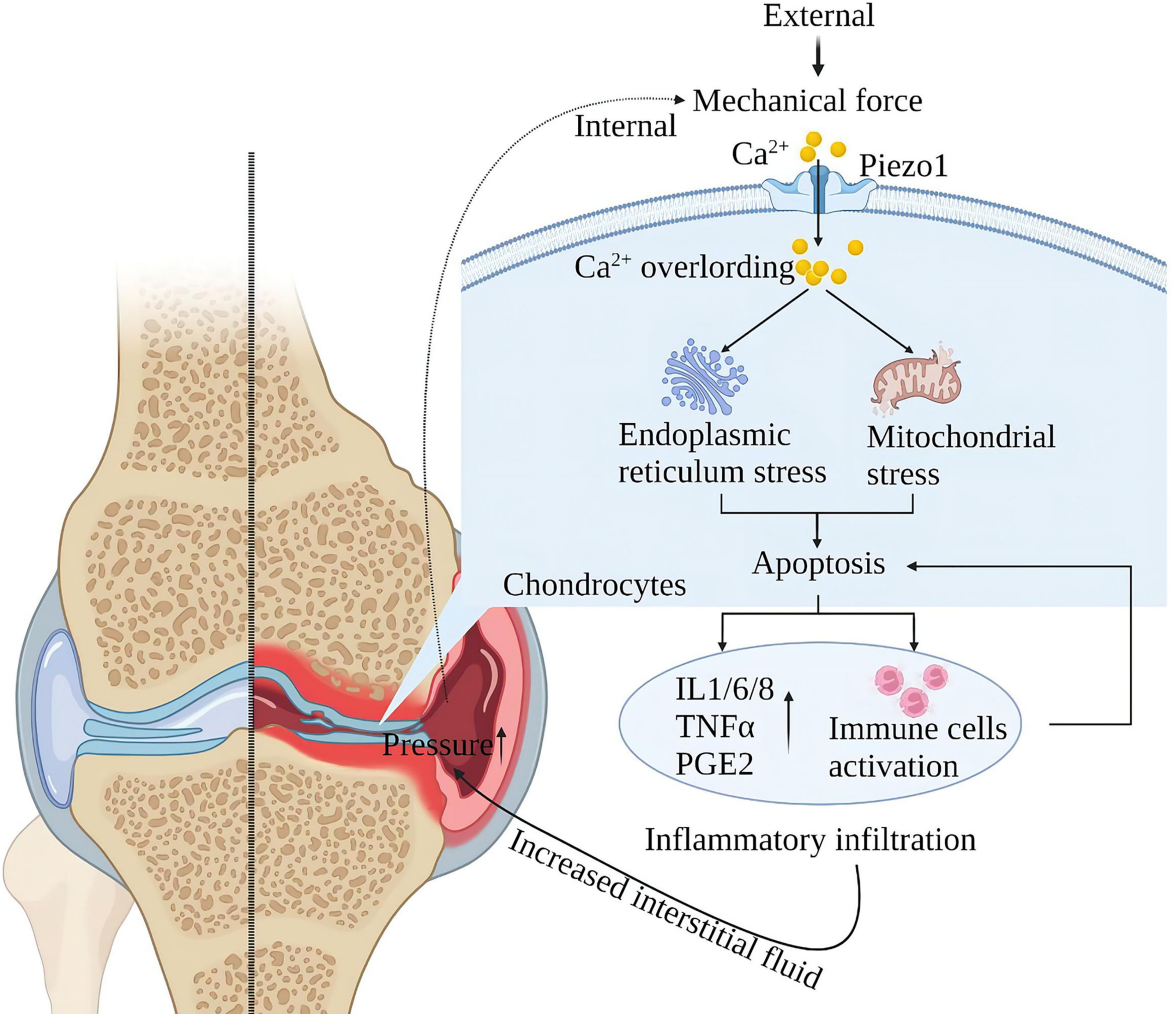
Schematic diagram of the role of Piezo1 in OA. Mechanical force activates Piezo1 on articular cartilage cells, induces Ca^2+^ overload, endoplasmic reticulum, and mitochondrial stress, leading to inflammation and apoptosis of chondrocytes. Cartilage cell damage leads to exudation of interstitial fluid and inflammatory infiltration. Inflammatory infiltration damages the nascent cartilage tissue, and exudation leads to increased intra-articular pressure, further activating Piezo1, creating a vicious injury cycle.

Piezo1 is a major skeletal mechanosensor that regulates skeletal homeostasis. Piezo1 plays an essential role in skeletal growth and development by affecting osteoblast-osteoclast crosstalk in response to mechanical forces (87–92). Mechanical forces at the physiological level are the basis for normal bone and joint function, and excessive mechanical loading of the bones can lead to inflammation and degenerative degeneration. In osteoarthritis (OA), upregulation of chondrocyte Piezo1 expression under high strain mechanical stress consistently enhances Ca^2+^ signaling leading to apoptosis (30, 31). Furthermore, Other studies suggest that MAPK/ERK5 and MAPK/ERK1/2 are downstream signal molecules mediated by Piezo1 channels to mediate late excessive apoptosis of chondrocytes under high-strain stimulation (93). After chondrocyte apoptosis, it will stimulate the environment in the joint and produce a large amount of oxygen free radicals and inflammatory mediators (such as IL-1β, TNFα, PE, etc.) to damage the new cartilage tissue and blood vessels. Chondrocyte loss and chronic inflammatory infiltration increase interarticular fluid, exerting pressure on intra- and extra-articular structures, further activating mechanoreceptors and injury receptors and creating a vicious cycle (94) (**Figure 8**).

**Figure 9:**
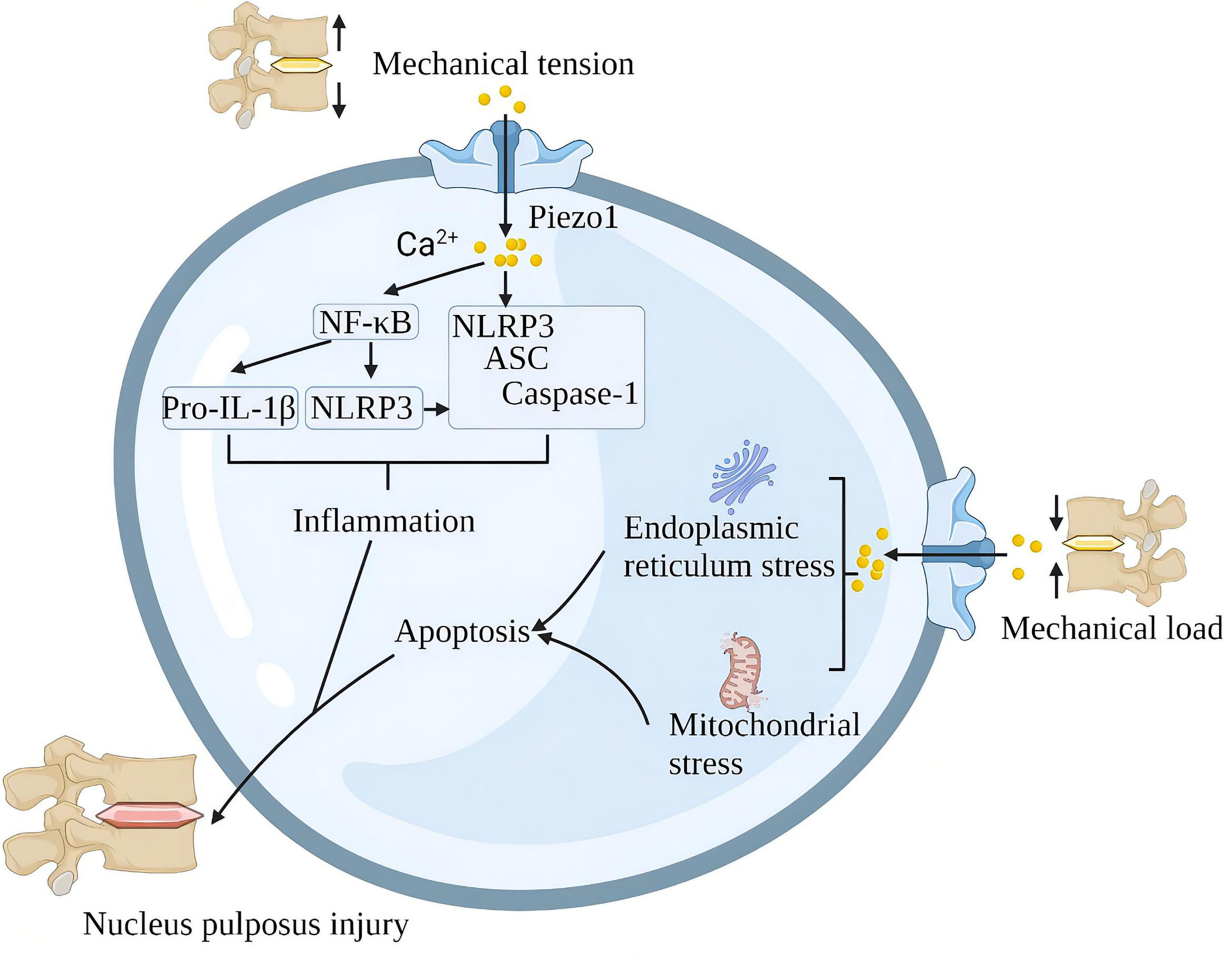
Schematic diagram of the role of Piezo1 in spinal degeneration. Piezo1 on the nucleus pulposus cells senses mechanical stretching and induces Ca^2+^ influx, which initiates the inflammatory pathways of NF-κB and NLRP3. Mechanical shock induces mitochondria and endoplasmic reticulum stress through Piezo1, leading to apoptosis of nucleus pulposus cells. Chronic inflammation and loss of nucleus pulposus cells lead to spinal degeneration.

A single impact applied to the lumbar disc without causing structural damage to the lumbar spine also triggered significant upregulation of Piezo1, NLRP3 inflammatory vesicles, catabolic (MMP-9, MMP-13) and pro-inflammatory gene (IL-1β) expression and induced disc degeneration (IDD). Piezo1 inhibition reduces mechanical shock-induced activation of NLRP3 inflammasome and IL-1β expression in nucleus pulposus cells (95). In addition, G protein-coupled estrogen receptor attenuates mechanical stress-mediated apoptosis of chondrocytes in osteoarthritis *via* suppression of Piezo1 (96). Mechanical stretching of the lumbar spine upregulates Piezo1, NLRP3, ASC and IL-1β in nucleus pulposus cells. In terms of mechanism, Piezo1 activation increases the cytoplasmic Ca^2+^ load, and Ca^2+^ activates the NF-κB pathway and increases the expression of NLRP3, proIL-1β and proIL-18. Ca^2+^ further stimulates inflammasome assembly, enhances the activation of caspase-1, and promotes the secretion of IL-1β (66). In the intervertebral disc, the rigid ECM around the nucleus pulposus tissue activates the Piezo1 channel of the nucleus pulposus cells to increase intracellular reactive oxygen species (ROS) and the expression of GRP78 and CHOP, which contributes to oxidative stress and endoplasmic reticulum stress. The continuous mechanical stress damage of the intervertebral disc leads to the death of cartilage cells, the reduction of extracellular matrix, the increase of various inflammatory factors, and finally, progress to lumbar degeneration (97) (**Figure 9**).

### Obesity and Diabetes

**Figure 10:**
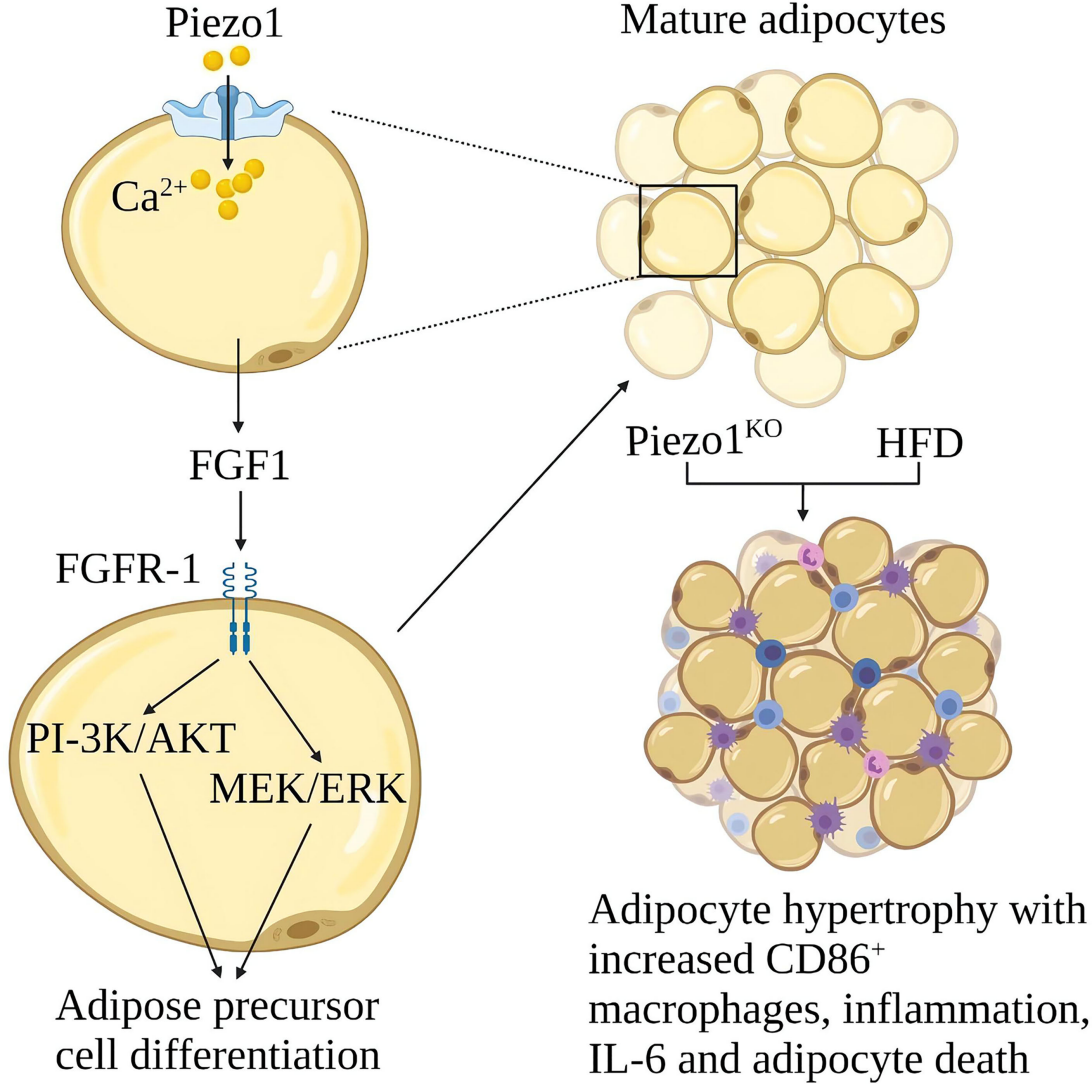
Schematic diagram of the role of Piezo1 in obesity and insulin resistance. Opening Piezo1 on mature adipocytes promotes FGF1 secretion, which induces adipose precursor cell differentiation. Mice lacking Piezo1 in adipocytes developed adipose progenitor cell differentiation disorder and increased hypertrophy after a high-fat diet, with increased CD86^+^ macrophages, inflammation, IL-6, and adipocyte death.

Obesity is due to excessive calorie intake, which leads to an increase in the size of fat cells (hypertrophy) and the number of cells (hyperplasia) (98). Hypertrophy promotes adipose tissue inflammation and insulin resistance. Proliferation leads to smaller fat cells, less adipose tissue inflammation and better insulin sensitivity (99). Piezo1 on the fat cell membrane can sense membrane tension from the inside out. The function of Piezo1 is essential for systemic lipid metabolism and insulin sensitivity (100). Piezo1 opening in developing mature adipocytes leads to the release of FGF1, which induces the Differentiation of adipocyte precursors through the activation of FGF-receptor-1, suggesting that Piezo1 opening is a signal that promotes the Differentiation of adipocyte precursors (101). Piezo1 -/- mice showed defects in the differentiation of adipocyte precursors into mature adipocytes when fed a high-fat diet (HFD), increased number of hypertrophic adipocytes, increased white adipose tissue (WAT) inflammation, and obesity, and reduced insulin sensitivity (9). The expression of Piezo1 was significantly increased in the white adipose tissue of obese mice, the mechanism of Piezo1 in obesity and insulin resistance needs further study (9) (**Figure 10**).

### Chronic Cystitis

**Figure 11:**
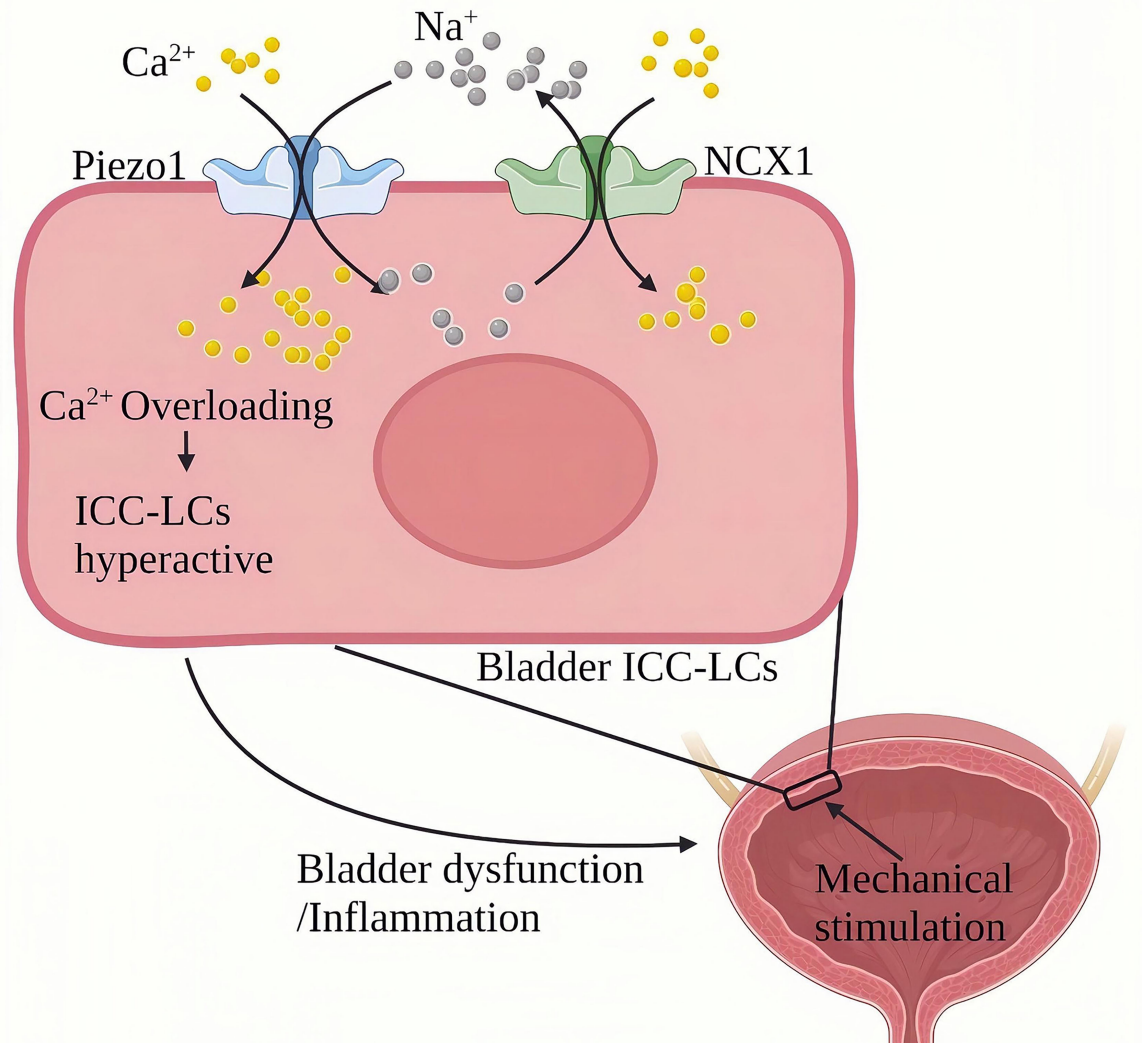
Schematic diagram of the role of Piezo1 in chronic bladder inflammation. The ICC-LC in the bladder senses mechanical stimulation and works in reverse with NCX to trigger the continuous flow of Ca^2+^ into the ICC-LC, leading to Ca^2+^ overload and ICC-LC dysfunction and inducing bladder function abnormalities and inflammation.

Chronic cystitis mainly manifests as chronic inflammation infiltration of the bladder and severe urinary frequency, urgency, lower abdominal pain, painful urination, hematuria and other symptoms (102). Piezo1 is widely expressed in the urinary system (103, 104). Qian Liu et al. (105)found that the expression of Piezo1 in bladder Cajal interstitial-like cells (ICC-LC) in the urothelium and mesenchyme of the cystitis rat model induced by cyclophosphamide (CYP) for 48 hours was significantly increased. CYP treatment for eight days resulted in a more significant up-regulation of Piezo1 in chronic bladder inflammation. ICC-LCs plays a vital role in regulating bladder activity, The abnormal number and function of bladder ICC-LCs are related to bladder inflammation (106–108). In terms of mechanism, Piezo1 and NCX reversely cooperate to trigger an increase in the continuous influx of Ca^2+^ into the bladder ICC-LCs. In bladder ICC-LCs, Na^+^/Ca^2+^ exchanger (NCX)-mediated ion transport is critical to cytoplasmic Ca^2+^ homeostasis in bladder ICC-LCs. NCX usually maintains Ca^2+^ homeostasis by removing Ca^2+^ from the cell forward and bringing Ca^2+^ into the cell in a reverse mode (109). In rats with chronic cystitis, the NCX reverse mode in the bladder ICC-LCs were relatively activated and cooperated with Piezo1. During chronic cystitis, mechanically activated Piezo1 promotes the flow of Ca^2+^ and Na^+^ into ICC-LCs. On the other hand, Piezo1 and NCX1 work in reverse to pump large amounts of Ca^2+^ to cause cellular Ca^2+^ overload and induce hyperfunction of the bladder (**Figure 11**). SiRNA targeting Piezo1 significantly reduced the reverse mode NCX1 on the ICC-LCs membrane and the increased Ca^2+^ and Na^+^ in the cytoplasm. GsMTx4(an inhibitor of Piezo1) can significantly improve the urodynamic abnormalities related to cystitis, significantly reduce the maximum bladder pressure (MBP), significantly extend the systolic interval (ICI), and relieve the symptoms of cystitis.

## Mechanism of Piezo1 Transducing Inflammatory Signals

The cytoplasmic Ca^2+^ under resting conditions is ∼10^-7^M, 10^4^ times lower than Ca^2+^ in the extracellular milieu (∼10^-3^M), various cell stimulations can promote the increase of cytoplasmic Ca^2+^ (110). Cytoplasmic Ca^2+^ mainly comes from extracellular and intracellular Ca^2+^ storage (endoplasmic reticulum (ER) and sarcoplasmic reticulum in muscle cells) (111, 112). The intracellular Ca^2+^ and Ca^2+^ signals must be strictly controlled. Ca^2+^ is the second messenger in the cell, and Ca^2+^, including inflammation, controls every cell aspect.

Based on the localization of Piezo1 in the cell, Piezo1 activation would not only trigger extracellular Ca^2+^ inward flow, but Piezo1 also promotes Ca^2+^ release from the Ca^2+^ pool (113). María Velasco-Estevez found that in the presence of extracellular Ca^2+^, Yoda1(an activator of Piezo1) initially enhances intracellular Ca^2+^ levels, most likely through the membrane-bound Piezo1 channel through Ca^2+^ influx, which may cause calcium-induced calcium release process (CICR) from intracellular stores (81). Fam38A mediates integrin activation by recruiting the small GTPase R-Ras to the ER, which activates the calcium-activated protease calpain by increasing Ca^2+^ release from cytoplasmic stores (18). The consumption of Ca^2+^ in the internal storage can activate the Ca^2+^ release-activated channel (CRAC) in the outer membrane, thereby enhancing the influx of extracellular Ca^2+^ and promoting the replenishment of the internal Ca^2+^ storage (114). Upregulation of Piezo1 in pancreatic acinar cells can induce PLA2 to activate TRPV4 channels and cause a continuous increase in Ca^2+^ (115). Hamza Atcha’s study (61)showed that the positive feedback regulation between Piezo1 and cytoskeletal actin promotes the activation of macrophage inflammation. Piezo1 enhanced the formation of F-actin, and actin polymerization promoted Piezo1-mediated Ca^2+^ activity, indicating a potential positive feedback regulation between ion channel activity and cytoskeleton. The transient or continuous increase of Ca^2+^ breaks the Ca^2+^ homeostasis, and the enhancement of the Ca^2+^ signal induces inflammation and other cellular events through a signal cascade.

The function of Piezo1 and its downstream mechanism have obvious differences in various tissue inflammations. Piezo1-mediated Ca^2+^ signaling participates in the occurrence and development of inflammation through a variety of signaling pathways (**Table 1**), including the classic inflammation pathway Jak/Stat: IL-6 receptor family (56, 120), TLR (9, 42) and NF-κB (66, 117, 121); Inflammasome NLRP3 (66, 95); Ca^2+^-sensitive MAPK family (ERK, JNK, P38) (116, 120, 122, 123), AKT/mTOR (60, 69)、β1 integrin (18); As well as focal adhesion kinase (FAK) related to inflammation-related mechanical transformation (60, 124), proline-rich tyrosine kinase2 (Pyk2) (125); Calcineurin (RACK1), calcium/calmodulin-dependent protein kinase II (CaMKII-Mst1/2-Rac) and calpain1 (18). Although the downstream mechanisms involved in Piezo1 are different in different organizations and occasions, they all eventually lead to inflammatory events.

**Table 1:** Current mechanisms are linking Piezo1 to the development and progression of chronic inflammatory diseases.

Diseases	Study	Mechanical Factors	Variation ofPiezo1	Animals/Target cells	Related Mechanisms	Piezo1 Inhibition
AD	(113)	Stiff amyloid plaques/LPS	Upregulate	Rat/Astrocyte	Intracellular Ca^2+^ oscillations	Decreased of inflammatory factors and chemokines
AD	(40)	Stiff amyloid plaques	Upregulate	Rat	The decoupling of neurons from astrocytes.	Promote decoupling of neurons from astrocytes
AD	(44)	Hyperglycemia /Hypertonic	Upregulate	Mouse/Microglia	Regulates Ca^2+^-related mTOR, JNK1 pathway	Improves microglia function
MF	(116)	Mechanical load	Upregulate	Human/Mouse /Cardiac Fibroblasts	p38 MAP-kinase signaling/IL-6	Reduces basal IL-6 expression
MF	(56)	Mechanical stretch	Activating	Mouse	Mediates Ca^2+^ and ROS signaling	Reduced stress damage
AS	(117)	Shear force	Upregulate	Mouse/Vascular endothelial cells	Gq/G11-mediated integrin activation induces focal adhesion kinase-dependent NF-κB activation	Inhibition of Piezo1 reduces inflammation and AS progression
Lung injury	(33)	Mechanical stretch	Upregulate	Rat/Human /Pulmonary microvascular endothelial cells	Promote RhoA/ROCK1 signaling	GSMTx4 alleviates the pathological changes in the lungs.
Lung injury	(83)	Mechanical stretch	Upregulate	Rat/Pulmonary microvascular endothelial cells	Up-regulation of Bcl-2	Knockdown of Piezo1 reduced apoptosis
IPF	(85)	Circulating hydrostatic pressure	Upregulate	Mouse /Macrophages	AP-1 drives c-JUN and endothelin 1 to stabilize HIF1α. Increase the secretion of chemokine CXCL2 in monocytes	Reduced lung inflammation
IDD	(66)	Mechanical stretch	Upregulate	Human/Nucleus pulposus tissue	Through the Ca^2+^/NF-κB pathway	Reduces the expression of NLRP3, ASC and IL-1β
IDD	(118)	ECM hardness	Upregulate	Human/Nucleus pulposus tissue	Increased intracellular ROS levels and the expression of GRP78 and CHOP	Reduces oxidative stress
OA	(93)	compression stress	Upregulate	Human /chondrocytes	MAPK/ERK5 and MAPK/ERK1/2 signal molecules	Inhibit the late stage of apoptosis
OA	(119)	Mechanical deformation	Upregulate	Pig/Human /Chondrocyte	P38 MAP-kinase and transcription factors HNF4 and ATF2/CREBP1.	Improving inflammation
Chronic cystitis	(105)	Mechanical stretching/Hypotonic	Upregulate	Rat/Bladder ICC-LC	Synergy of Piezo1 and reverse mode NCX1 leads to cytoplasmic Ca^2+^ overload.	Improves ICC-LC hyperfunction and chronic cystitis

IPF, idiopathic pulmonary fibrosis.

## Piezo1 and Chronic Inflammatory Pain

Chronic inflammation is often accompanied by physical and psychological symptoms, including pain and mood changes. Pain is an adaptive response of the body to injury, and these symptoms have a serious impact on the quality of life during chronic inflammation (126). Nociception provides a means of neurofeedback that allows the central nervous system (CNS) to detect and avoid toxic and potentially destructive stimuli (for example, heat, cold, mechanical, or chemical stimuli) inactive and passive environments (127).The accumulation of K^+^ and H^+^ in local lesions during inflammation, especially inflammatory mediators such as prostaglandin, serotonin, bradykinin, and other stimulating nociceptors, is the main cause of pain (127). Exudation in the inflammatory lesion causes tissue swelling, increased tension, and compression of nerve endings can cause pain. In addition, the inflamed organ enlarges, which increases the tension of the capsule rich in sensory nerve endings, and the nerve endings are stretched and cause pain. Cytokines can also affect CNS activity through various body fluids and neural communication pathways (such as the vagus nerve afferent nerve). Recent studies have suggested that many C-type nerve fibers feel strong stimulation under normal conditions but do not cause pain. However, under the action of inflammatory mediators, these nociceptive fibers are more sensitive to mechanical stimuli, even if they are mildly stimulated. It can also cause pain.

The research of Piezo1 channel in pain is still in the early stage. Piezo2 has been confirmed to be related to various pain (128). Piezo1 and Piezo2 channels are both expressed in sensory neurons. Piezo2 is highly expressed in dorsal root ganglion neurons of various sizes; the most prominent is a giant diameter neuron involved in mediating touch and proprioception. In contrast, Piezo1 is selectively expressed in smaller DRG neurons that mediate nociception, indicating its potential role in pain (129). Knockout of Piezo2 in the same mouse sensory nerve will damage the sense of touch, but will make the mice sensitive to mechanical pain, indicating a negative interaction between Piezo1 and Piezo2.

Recently, Piezo1 was confirmed to be expressed in the trigeminal ganglion and is involved in the mechanical transduction of migraine. In ex vivo hemiskull preparation, Yoda1 activates the continuous nociceptive discharge of the meningeal branch of the trigeminal nerve to a large extent (130). Interestingly, Mingmin Zhang’s research results show that Piezo1extensive expression in sensory neurons will reduce rather than cause mechanical pain responses (131). Another study (132) showed that Piezo1 was involved in mediating the reduction of pain threshold caused by sleep deprivation, while microinjection of GSMTx4 or PD151746(a calpain inhibitor) partially reversed the pain threshold. Therefore, the research of Piezo1 in pain still needs further exploration.

## Pharmacology and Physical Regulation of Piezo1 Channel

Inflammation-induced mechanical injury is mainly sterile, and antibiotic treatment is ineffective because there is no specific pathogen, such as bacterial infection (127). The treatment of aseptic inflammation should be tailored to the different etiologies. Based on understanding the pathogenesis of chronic tissue inflammation caused by mechanical force imbalance, reducing destructive mechanical force is important to prevent and treat chronic tissue inflammation. Many targeted preventive measures have been put forward clinically, and significant effects have been achieved. For example, joint braking and non-steroidal anti-inflammatory drugs are early treatments for osteoarthritis (133, 134). Patients with lumbar spine degeneration should rest in bed to relieve spinal pressure (135, 136). Stent implantation can correct local disordered hemodynamics and effectively delay the progression of atherosclerosis (62, 137). Piezo1 channel provides solid theoretical support for these clinical prevention measures. This also reminds us that for chronic inflammation caused by mechanical damage, a good lifestyle is better than all treatments after inflammation (138, 139). However, most chronic tissue inflammation has an insidious onset, and damaging mechanical signals are usually long-standing before clinical symptoms. Therefore, Piezo1 pharmacology is of particular importance.

Currently, only few drugs are used in pharmacological studies of the Piezo1 channel (**Table 2**). They include lanthanides Gd^3+^ and La^3+^, aminoglycoside antibiotics (such as streptomycin and gentamicin), Ruthenium red (RR) (16), and GsMTx-4 peptide isolated from tarantula spider toxins (143). They block most MSCs, including Piezo1, and inhibit MA currents induced by Piezo1 (157). Mechanistically, GsMTx4 can bind to the cell membrane. In the resting state, GsMTx4 is stabilized by six lysine residues on the surface. When the membrane tension increases, the lateral pressure in the phospholipid bilayer decreases, and the penetration distance of GsMTx4 on the cell membrane increases, which in turn causes the outer layer of phospholipid molecules to relax locally, thereby reducing the efficiency of force transduction from the lipid bilayer to the channel (143). Another class of compounds, such as amphiphilic chlorpromazine and LPC, has been shown to activate prokaryotic and eukaryotic MSCs (158, 159). Yoda1 is a new class of synthetic small molecule compounds that can specifically activate the Piezo1 channel. Yoda1 at a micromolar concentration induces a strong Ca^2+^ response in cells transfected with human or mouse Piezo1 (140). Jedi1/2 is a new type of hydrophilic Piezo1 chemical activator, which acts through the peripheral blades and uses a peripheral lever-like device composed of blades and light beams to gate the central ion conduction hole (142). Recently, an analogue of Yoda1, named Dooku1 (147), and tubeimoside I (TBMS1) identified in traditional Chinese medicine (148), reversibly antagonizes the activation of Piezo1 induced by Yoda1 through competition for specific channel binding sites.

**Table 2:** Currently reported drugs and physical methods can regulate Piezo1.

Category	Drugs	Drug properties	Selectivity	In vivo evidence for Piezo1
Activatior	Yoda1 (140)	Synthetic compound	Selective	Rat/Intracranial injection (132). Mouse/Intravenous injection (141)
	Jedi1/2 (142)	Chemical activator	Selective	None
Inhibitor	GsMTx4 (143)	Peptide toxin	Nonselective	Rat/Intracranial injection (132)/Arterial injection (33). Mouse/intravenous injection (141).
	Ruthenium red(RR) (19)	A polycation	Nonselective	None
	Gd^3+^ (144)	Rare earth ion	Nonselective	None
	Enantiomeric amphipathic Aβ peptides (145)	Peptide	Nonselective	None
	Neutral sphingomyelinase inhibitors (146)	Compound	Nonselective	None
	Dooku1 (147)	Compound	A specific antagonist of yoda1	None
	Tubeimoside I (148)	Traditional Chinese Medicine	Nonselective	None
	Phosphatidylinositol 4,5-bisphosphate (149)	Compound	Nonselective	None
Modifier	Capsaicin (149)	Compound	Nonselective	/
	Margaric acid(saturated) (150)	Fatty acid	Nonselective	None
	Polyunsaturated fatty acids (unsaturated) (150)	Fatty acid	Nonselective	None
	Cholesterol (151)	Lipid	Nonselective	/
Physical	Ultrasound (152, 153)	/	Nonselective	/
	Electricity (154)	/	Nonselective	/
	Ionizing radiation (155)	/	Nonselective	/
	Magnetic energy (156)	/	Nonselective	/

The activity of the Piezo1 channel can also be adjusted physically. With the advantages of high resolution and good penetration, ultrasound technology has been widely used in clinical practice. In 2018, the Maduke research team discovered that the Piezo1 could be activated by 43MHz ultrasound outside the body to become open (160). A shock wave (SW) is a mechanical energy wave generated by the medium’s highly rapid compression and accumulation through vibration or high-speed motion (161, 162). The mechanical stress generated by the extracorporeal shock wave stimulates the cells to make the cell membrane structure elastically deform, activate the mechanosensitive ion channel protein on the cell membrane, and produce a series of biological effects (163, 164). Electricity plays a vital role in wound healing by activating Piezo1 channels and Ca^2+^ influx (154). Knockdown of Piezo1 partially reversed radiation-induced in vitro epithelial-mesenchymal transition and played a therapeutic role in bleomycin-induced pulmonary fibrosis in rats (155). It also suggests the potential role of Piezo1 in tumor radiotherapy. Magnetic nanocomposite materials can activate Piezo1 to induce osteoblast differentiation and promote bone repair (156). Mechanical forces are the main mechanism for activation of Piezo1 channels. The use of physical methods to control the expression of Piezo1 has excellent clinical application prospects (**Table 2**).

## Conclusions and Future Research Directions

As a newly discovered mechanically sensitive ion channel, Piezo1 plays a vital role in inflammation triggered by mechanical stress imbalance and changes in the local environment of cells. Piezo1 transduces mechanical damage signals into intracellular inflammatory cascades to drive inflammation. Changes in local pressure during the progression of inflammation may further affect the outcome of inflammation through Piezo1 (165, 166). Mechanical stress and inflammation are both dynamic processes. The link between Piezo1 and inflammation is a growing field that helps us understand how individual cells respond to the environment’s continuous and diverse mechanical stimuli.

The discovery of Piezo1 provides new ideas for the treating chronic inflammatory diseases. Inhibiting the transduction of mechanical damage signals at an early stage can reduce the incidence and improve the outcome of chronic inflammation. Although the function of Piezo1 in inflammation has been established, its pharmacological effects are still limited to low-affinity drugs (activators and blockers) with poor solubility and stability. They cannot be used in vivo (167). Therefore, more work is needed to determine more modifiers.

When Piezo1 is used as a treatment strategy, understanding how tissue-specific factors regulate the sensitivity of Piezo1 channels will improve the specificity of any potential treatment. For example, in AS, inhibition of Piezo1 is not the best way to fight AS because it brings about vascular tension and hypertension (168). In addition, some diseases are not caused by changes in the Piezo1 channel’s state but by changes in the Piezo1 channel itself (169, 170). Therefore, Piezo1 drugs with tissue specificity may be the dawn of future disease treatment, and the development of drugs targeting the downstream pathway of Piezo1 is also an alternative approach. These strategies may help reduce disease and treatment costs under mechanical stress, especially chronic inflammatory diseases.

Similar to Piezo1, Piezo2 is a newly discovered mechanosensitive ion channel. Piezo2 has a similar structure to Piezo1 as well as mechanosensitivity (171). Piezo2 is expressed on and exerts mechanotransduction in various mechanosensitive tissues and is associated with various inflammatory diseases (172–174). Human Piezo2 defects and Piezo2 gain-of-function mutations are associated with respiratory insufficiency at birth, chronic obstructive pulmonary disease, and adult sleep apnea (175–177). Piezo2-deficient mice cannot respond to the punctate and dynamic allodynia induced by capsaicin-induced inflammation and nerve damage, indicating that Piezo2 mediates sensitized mechanical pain caused by inflammation and nerve damage (178). Piezo2 may also be a therapeutic strategy for chronic inflammation induced by mechanical stress. Piezo1 and Piezo2 also have signal crosstalk. Piezo1 and Piezo2 are highly expressed in articular chondrocytes and jointly promote Ca^2+^ signal transduction in chondrocytes caused by mechanical force (31). Inhibition of Piezo1 and Piezo2 reduces the death of cartilage cells after mechanical injury *in vivo*, but the mechanism of how Piezo1 and Piezo2 channels work together at the molecular level remains unclear.

In addition to the piezoelectric family, MSC also includes degenerations/acid-sensitive channels, TREK/TRAAK, transient receptor potential channels, mechanically sensitive channels of the TMEM16 superfamily, OSCA/TMEM63, these ion channels can sense various forms of force, and Conversion of mechanical stimuli into electrical or chemical signals (179). Some ion channel-mediated signals are also active participants in inflammation (180–182). In the acinar cells of patients with acute pancreatitis, the Piezo1 of the acinar cells is upregulated and induced PLA2 to activate the TRPV4 channel to cause the continuous increase of Ca^2+^ and damage the mitochondrial function (115). Piezo1 responds to long-term shear stress and regulates the activation of TRPV4 channels in vascular endothelial cells, resulting in continuous Ca^2+^ inflow (183). The connection between Piezo1 and other MSCs deserves further study.

In short, as a sensory component of mechanical signals, the relationship between Piezo1 channels and inflammation is precise and complex. The discovery of Piezo1 provides a new direction for the treatment of diseases. It inhibits the occurrence and development of inflammatory events in the early stage after mechanical damage is an effective treatment for the prevention and treatment of chronic inflammatory diseases. Piezo1 pharmacology has shown promising prospects. The link between the Piezo1 channel and chronic inflammation and Piezo1 drugs for clinical application will focus on future research.

## Author Contributions

HL and JH performed the literature search and manuscript writing. ZQ edited and revised the manuscript. XF, FZ, and XW edited the manuscript. GX and FH guided and revised this manuscript. All authors have read and agreed to the published version of the manuscript.

## Funding

This work was supported by grants from the National Natural Science Foundation of China (82060219, 81860259); Natural Science Foundation of Jiangxi Province (20202BAB206033 and 20202BABL206016); Youth Team Project of the Second Affiliated Hospital of Nanchang University (2019YNTD12003).

## Conflict of Interest

The authors declare that the research was conducted in the absence of any commercial or financial relationships that could be construed as a potential conflict of interest.

## Publisher’s Note

All claims expressed in this article are solely those of the authors and do not necessarily represent those of their affiliated organizations, or those of the publisher, the editors and the reviewers. Any product that may be evaluated in this article, or claim that may be made by its manufacturer, is not guaranteed or endorsed by the publisher.
